# Nanoporous-Gold-Based Hybrid Cantilevered Actuator Dealloyed and Driven by A Modified Rotary Triboelectric Nanogenerator

**DOI:** 10.1038/srep24092

**Published:** 2016-04-11

**Authors:** Xuequan Li, Mengmeng Liu, Baisheng Huang, Hong Liu, Weiguo Hu, Li-Hua Shao, Zhong Lin Wang

**Affiliations:** 1 Beijing Institute of Nanoenergy and Nanosystems, Chinese Academy of Sciences; National Center for Nanoscience and Technology (NCNST), Beijing 100083, P. R. China; 2 School of Material Science and Engineering, Georgia Institute of Technology Atlanta, Georgia 30332–0245, USA

## Abstract

We firstly designed an electrochemical system for dealloying to synthesize nanoporous gold (NPG) and also driving the novel NPG based actuator by utilizing a modified rotary triboelectric nanogenerator (TENG). Compared to the previous reported TENG whose outputs decline due to temperature rising resulting from electrodes friction, the modified TENG with a cooling system has stable output current and voltage increased by 14% and 20%, respectively. The novel cantilevered hybrid actuator characterised by light-weight (ca. 3 mg) and small volume (ca. 30 mm × 2 mm × 10 μm) is driven by a microcontroller modulated TENG with the displacement of 2.2 mm, which is about 10^6^ times larger than that of traditional cantilever using planar surfaces. The energy conversion efficiencies defined as the energy consumed during dealloying and actuation compared with the output of TENG are 47% and 56.7%, respectively.

Actuators refer to the devices that can reversibly change their dimensions upon the application of an external stimulus, such as an applied voltage^1^. The electrical actuator regulated by an applied electrical signal is mostly investigated owing to its better controllability, higher energy conversion efficiency and larger strain^2^. In the recent years, nanoporous gold (NPG) serving as an electromechanical actuator has attracted much interest. The high surface-to-volume ratio of NPG gives rise to the large volume changes, high stiffness and strength^1^,^3^,^4^,^5^,^6^,^7^,^8^. NPG is normally fabricated by dealloying – a selective removal of Ag from an alloy AuAg solid solution by applying a suitable voltage in an electrolyte^9^,^10^,^11^ – which results in a gold solid skeleton with interconnected pores or voids at nanometer scale^12^. Both of the fabrication of NPG and driving the actuators require external power source^1^,^13^.

Recently, Triboelectric nanogenerator (TENG) attracts great attention since it is a promising green energy source by converting waste mechanical energy into electricity at an efficiency as high as 55%^14^,^15^,^16^,^17^. The working principle of TENG is based on the combination of contact electrification and electrostatic induction through the contact and separation or relative sliding between materials with opposite tribopolarity. The charge transfer between these two tribomaterials results in a potential difference, which will drive the electrons/ions to flow in the external circuit. The equivalent circuit model of TENG can be represented as a combination of a capacitor and an ideal voltage source^18^. Their resistive load characteristics depends on the impedance match mechanism^19^. Many types of TENG have been proposed intensively, among them, the rotary TENG has shown advantages of high energy conversion efficiency, low cost, and abundant choices of materials^11^. TENG has been successfully applied for electrochemical purposes, such as water splitting, electrodeposition and anti-corrosion^20^,^21^,^22^. Hitherto, both of the voltage and current outputs of TENG are still impulse after rectification, more complicated electrochemical activities powered by TENG are still unknown such as dealloying and actuation. Meanwhile, the friction between two electrodes of a rotary TENG makes thermal energy, and the increasing temperature will decrease the output power of TENG in an isolated system^23^. To the best of our knowledge, the dependence of the output of the rotary TENG on temperature and how to decrease the temperature to enhance the output and working time have not been investigated yet. It is worth noting that all above mentioned issues will be great challenges of TENG for practical application as a stable and sustainable green power source compared to the traditional power source.

In this work, we firstly designed an electrochemical system for dealloying to synthesize NPG and also for driving the NPG based actuator by utilizing a motified rotary TENG with cooling system as a promising stable and sustainable green power source. The novel hybrid cantilevered actuator in this work has light weight, large movement, higher frequency, lower cost and fast response. It is confirmed that more complicated electrochemical activity such as dealloying and actuation can be powered by the modified TENG.

## Results

### The Outputs of TENG

**Figure 1:**
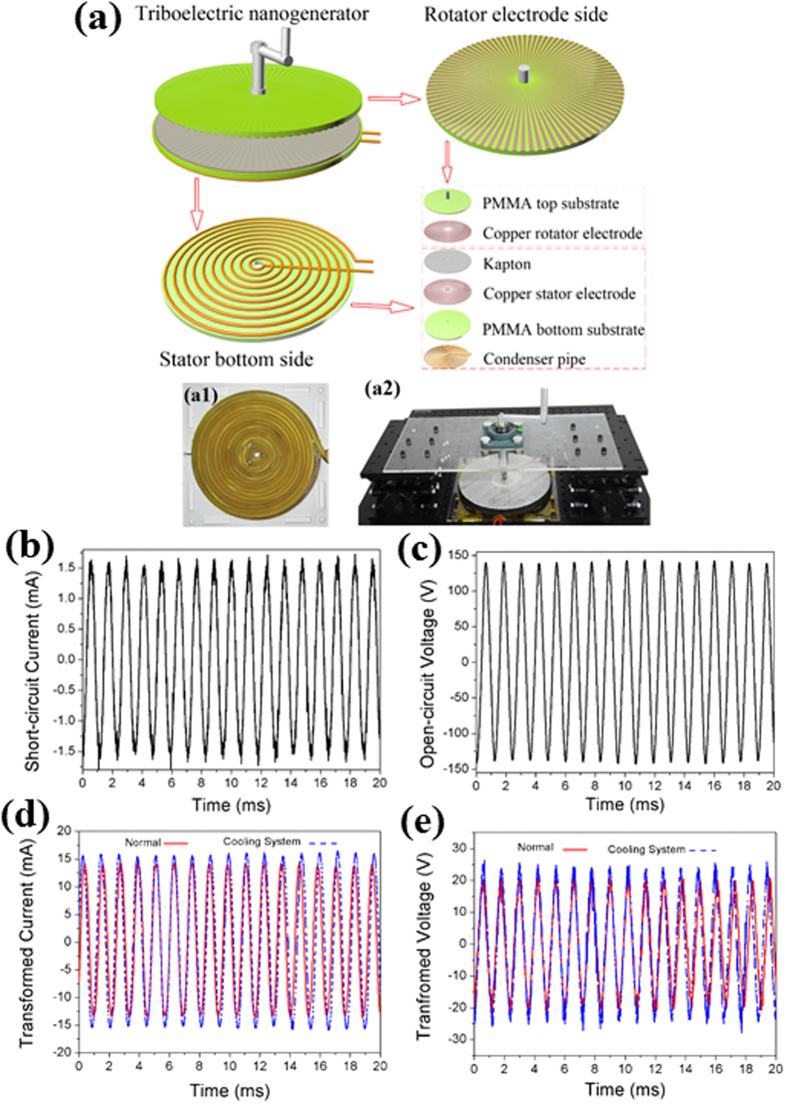
Structural design and characterizations of the disk TENG. (**a**) Schematic illustrations and the photographs of the disk TENG. (**b**) The short-circuit current and (**c**) the open-circuit voltage of the as-fabricated disk TENG. (**d**) The short-circuit current and (**e**) the open-circuit voltage after applying a transformer with (blue dashed curve) and without (red solid curve) the cooling system.

The TENG’s structure is schematically shown in Fig. 1(a). The working principle of the rotary TENG has been reported before^24^,^25^. Because of the 2D planer structure (small volume: the dimension of the disk is ca. 160 mm × 10 mm) and the lightweight of the rotator (ca. 400 g), the TENG could be easily driven by a manual rocker as shown in Fig. 1(a2). TENG can also harvest various mechanical energy such as wind, flowing water, oscillation driven by windmill, hydroturbine and so on^21^,^24^. The output current and voltage under the rotating speed at 200 rpm of TENG are shown in Fig. 1(b,c), respectively. A continuous AC output short circuit current, *I*, can be observed at an average amplitude of 1.5 mA. The open-circuit voltage (*V*oc) oscillates at the same frequency as that of *I* with an amplitude of ca. 140 V. The continuous AC output from TENG could be modulated by using the conventional transformer, which is effective to increase the output current by consuming the output voltage – which is also ideal for most of the electrochemical purposes – as shown in Fig. 1(d,e) (red solid curve). Ideally, the transformed current was enhanced up to ca. 14 mA, while the voltage was reduced to about 20 V. In order to enhance the working efficiency and the continuous-running duration of TENG, we firstly construct the condenser pipe cooling system as presented in Fig. 1(a1). The circulating cooling water is pumped into the pipe during the TENG rotating, and the resulted output current and voltage are increase by 14% and 20%, respectively, as shown in Fig. (d) and (e) (blue dashed curve). It has also been studied that the output power of TENG at low temperature is larger than that at high temperature^23^. However, the liquid nitrogen was used for cooling in that study, which is inconvenient and expensive for application. As shown in this work, the outputs of TENG have been significantly increased compared to the one without a cooling system whose outputs decline due to the temperature rising resulting from electrodes friction.

### The Working Current and Voltage

**Figure 2:**
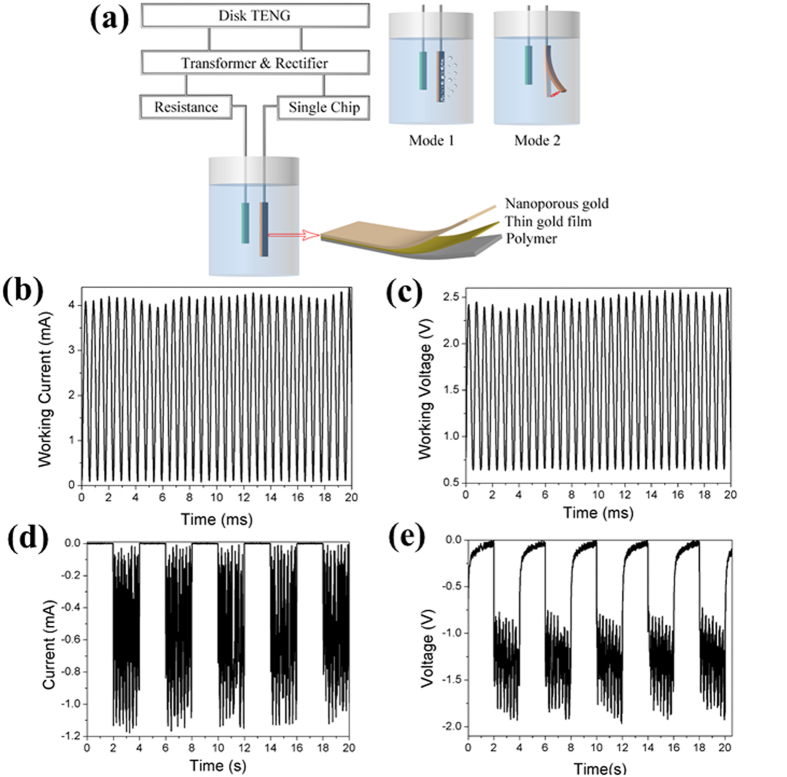
The working current and voltage of the electrochemical system for dealloying and actuation. (**a**) Schematic diagram of the electrochemical system for dealloying to synthesize nanoporous gold and to drive the hybrid actuator by the modified rotary TENG. (**b**) The working current and (**c**) voltage during dealloying. (**d**) The modulated output current and (**e**) voltage during actuation.

The electrochemical system is schematically shown in Fig. 2(a), where the TENG was subsequently connected to transformer, rectifier, resistor, single chip and the electrochemical cell by copper wires with alligator clips for two purposes, which were named as Mode 1 and Mode 2, respectively. The detailed equivalent circuit of TENG^18^ equipped with a transformer and a rectifier successively as a power source is demonstrated in supplementary Fig. S1. In Mode 1, the output electricity of TENG was used for dealloying–dissolving Ag from AuAg alloy in acid. The corresponding working current and voltage during dealloying are plotted in Fig. 2(b,c). The electrochemical dealloying was performed in a two electrodes setup: a positive voltage was continuously applied on the working electrode (alloy), and then the resulting positive current can be measured. The counter and reference electrodes were a platinum plate with the dimension of 1 cm × 1 cm. The original outputs were impulses at very high frequency (ca. 10^6^ Hz), hence, we took the average current and voltage of 2 mA and 1.5 V as the working current and voltage, respectively. To guarantee the dealloying process efficiently, the highest applied voltage was set to 2.5 V which may lead to some electrolyte decomposition. But the decomposition is not an issue to the reaction. In mode 2, the hybrid actuator was driven by TENG. A programmed single chip was connected to TENG to achieve switching on and off the circuit every two seconds. Thus, the output current and voltage are stepped as plotted in Fig. 2(d,e). The average working current and voltage are −0.5 mA and −1.2 V. The detailed procedure will be explained below.

### Electrochemical Corrosion Powered by TENG

**Figure 3:**
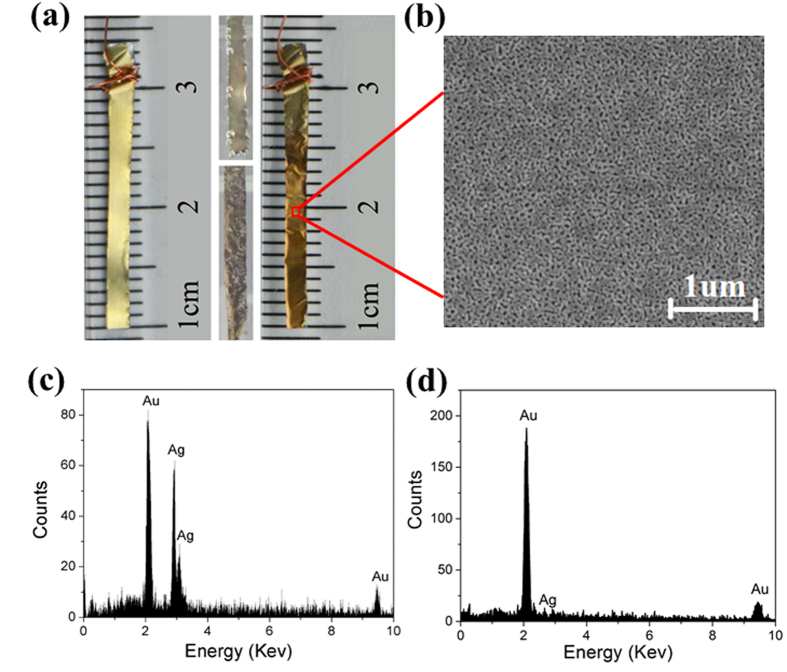
Characterization of the nanoporous gold (NPG) dealloyed by the output power of TENG. (**a**) The photographs of the top layer of the composite material before, during and after dealloying. (**b**) The SEM image of the NPG. The EDS spectra of the sample (**c**) before and (**d**) after dealloying.

The composition of hybrid cantilevered actuator was schematically demonstrated in Fig. 2(a) and the real sample consisting of polymer, gold film and alloy film was obtained as the left photograph shown in Fig. 3(a). The top alloy layer is golden color. The two photographs in the middle of Fig. 3(a) present the situation during dealloying, which is featured by color change and the emerging bubbles on the sample surface. The bubbles on the sample are O_2_ gas evolved during dealloying, which can be explained by the following equations:


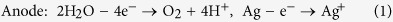



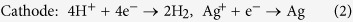


The hybrid cantilevered actuator mainly based on NPG is shown in the right photograph of Fig. 3(a). The color of the sample surface turns to much darker color, which is normal for NPG^26^. The hybrid actuator consists of three layers, namely, polymer, gold film and NPG. From the scanning electron microscope image of the top layer in Fig. 3(b), one can see that the ligaments and pores of NPG distribute uniformly with the feature size at around 50 nm. Figure 3(c,d) qualitatively give the composition of the sample before and after dealloying. One can find that nearly all of the Ag has been removed after dealloying by TENG, which means that TENG’s performance is comparable with potentiostat for electrochemical corrosion.

The mass of the top layer alloy, *m*_t_ is about 0.546 mg, if all of the Ag are dissolved then the transferred charge, *Q* should be around 282 mC (for detailed calculation please see Supporting Information). And if we assume the average output voltage, *V*_avg_ of TENG is equal to the working voltage, *V*_work_ of the sample during dealloying, the electric energy consumption, *W*_work_ of the Ag dissolving as Ag^+^ procedure is *W*_work_ = *V*_avg_*Q*. The output energy of TENG is *W*_output_ = *V*_avg_ *I*_avg_ *t*. The energy conversion efficiency of TENG, ζ is defined as ζ = *W*_work_/*W*_output_. Here, *I*_avg_ is 2 mA, *t* is 300 s. Therefore, the energy conversion efficiency of dealloying is up to 47% compared to the output of TENG.

### The Hybrid Cantilevered Actuator Powered by TENG

The hybrid cantilevered actuator in this work is an electrochemical actuator, the working principle can be simply described as: varying the applied voltage injects electronic charge into NPG electrode, and the change in charge density leads to the changing of surface stress. Since the pressure in the bulk required to balance the surface stress change, the large volume change of NPG can be obtained^5^,^27^. The electrochemical charging needs certain time to fully charge the whole surface of NPG. In order to obtain the largest strain, one needs to allow enough charging time. Thus the TENG’s outputs were on/off every two seconds controlled by the programmed microcontroller connected in the circuit as Fig. 2(a) schematically shown.

**Figure 4:**
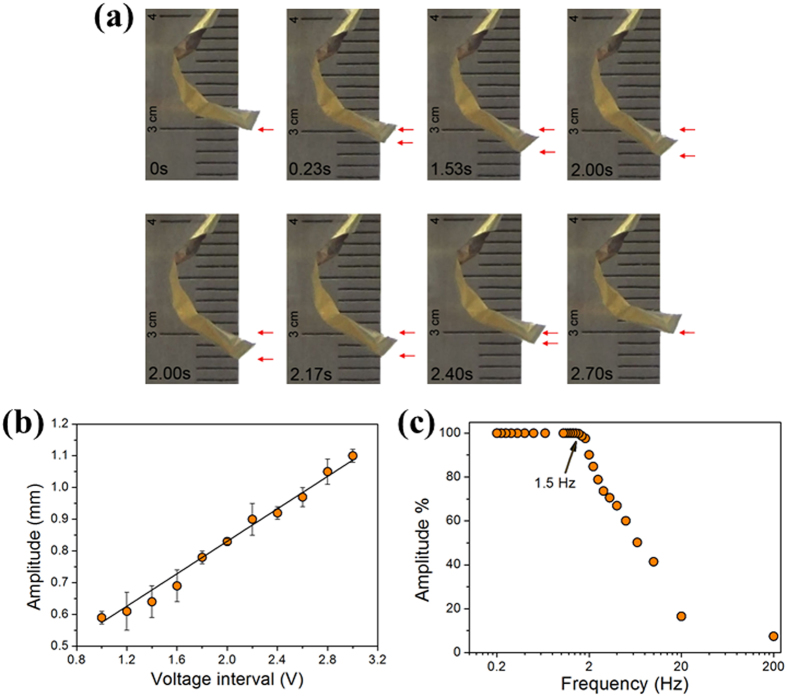
Characterization of the nanoporous gold based hybrid cantilevered actuator driven by the output power of TENG. (**a**) The photographs of the actuation procedure with one cycle driven by TENG. (**b**) The amplitude versus the applied voltage interval by function generator. (**c**) The frequency dependence of the amplitude (in the form of percentage of the maximum strain amplitude) during voltage jumps between −1 V and +1 V.

Here, the charge induced expansion or contraction of NPG leads to a biaxial stress component that resulting in a large bending of the composite foil (see the video in Supporting Information). Figure 4(a) are the photographs cut from the video, which presents the actuation procedure with one cycle driven by TENG. When the circuit was on, the output voltage (current) started to working – in the mean time the tip of the actuator moved downwards. As the red color arrow marked, the tip moves as much as ca. 2.2 mm, which is similar to the displacement of the actuator fabricated by NPG on gold foil^5^. This surface stress induced displacement is about 10^6^ times larger than that of the cantilever bending within the nanometer regime using planar surfaces. In Fig. 4, the sample is bended compared to the one shown in Fig. 3(a), which because that the NPG contract after dealloying compared with the alloy, which leads to the dimension decrease of NPG^12^. While the gold film and polymer layers are still at the original dimension, so the sample tends to bend. Especially when it is immersed in the electrolyte, the buoyance also makes the sample bending.

As one can see in Fig. 4(a), it takes 2 s for the tip moving down, while the tip moving up just uses 0.7 s. If we observe the video carefully, it is easy to see that there are two kinds of movements: one is the large displacement to 2.2 mm, the other is the small vibration only happened during the tip moving down, i.e. charging process. There are also two outputs periods of TENG: (1) The original outputs period of TENG is less than 1 ms as shown in Fig. 2(d,e), which is too short to fully charge NPG. Thus, the small vibration during charging might be the reaction of the charged top layer of NPG with the slower reaction than TENG’s original frequency. (2) The other outputs period of TENG is the artificial one, 4 s, i.e. 2 s charging and 2 s discharging processes. Within the charging process, the averaged voltage and current of TENG contributes to driving the actuator with large displacement. The longer of the charging time, the more electric energy converted to mechanical energy, i.e. the larger of the actuator’s displacement. While the discharging was finished instantly, since the output electricity was cut off immediately after the first 2 s.

For rough calculation we simplify the hybrid actuator as a cantilevered beam mainly consisting of Parylene, which is reasonable since the thickness of the polymer layer is far more than that of the gold and NPG layer. Then the work done, *W*_Act_ during the actuation is *W*_Act_ = ½ *F*Δ, where *F* is the total load on the beam and Δ = 2.2 mm is the deflection of the cantilever tip. Here 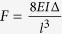 and the moment of inertia is 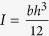
28, where *E* = 3.2 GPa is the modulus of elasticity of Parylene^29^, *l* = 12 mm the length immersed in the electrolyte, *b* = 10 μm the thickness and *h* = 2 mm the width of the beam. The work done during the small vibration, *W*_small_ should also be counted. We assume the total displacement is 0.2 mm within one cycle of the small vibration during charging, and there are about 10 cycles in 2 s. Thus, *W*_small_ = ½ *F*δ, where *F* is the total load on the beam and δ = 2 mm is the total deflection of the small vibration. Therefore the total mechanical work done within 4 s, *W*_t_ = 2*W*_Act_ + *W*_small_ is about 0.68 mJ, since the total deflection including the large displacements moving down/up and the small vibration (for detailed calculation please see Supporting Information). The output energy of TENG is *W*_outputAct_ = *V*_avgAct_*I*_avgAct_*t* = 1.2 V * 0.5 mA * 2 s = 1.2 mJ. The energy conversion efficiency of TENG for actuation, ζ = *W*_t_/*W*_outputAct_ is as high as 56.7% compared to the output power of TENG.

In order to quantitatively characterize the performance of the hybrid actuator, a function generator was used as power source. The stepped voltage using 0 V as the midpoint was applied on the sample, and the magnitudes of the voltage interval were recorded. The resulting displacement amplitude versus voltage is plotted in Fig. 4(b), which shows that the amplitude changes linearly with the applied voltage. Figure 4(c) shows the frequency dependence of the amplitude (in the form of percentage of the maximum strain amplitude) during voltage jumps between −1 V and +1 V. The characteristic frequency can achieve up to 1.5 Hz, which is 3 times larger than that of the actuator reported in ref. 5. In contrast to the NPG on gold actuator, the hybrid actuator in this paper is inexpensive, light-weight, and reacts much faster, since the gold foil substrate is replaced by polymer. Besides, this characteristic frequency of 1.5 Hz is corresponding to the actuation time of 0.67 s, which agrees well with the actuator moving up duration, 0.7 s, as shown in Fig. 4(a).

## Discussion

Since the rotary TENG can convert different mechanical energy into electricity, such as wind, flowing water and human activity, the electrochemical system can be used in various environment. For instance, in some special cases it is hard to connect with an external power source. Or for wearable devices, one can take advantage of the system to transfer the human activity into energy to power the sensors or other devices.

The novel light-weight cantilevered actuator, which exhibited large displacement, faster response and lower cost compared to the reported NPG on gold foil^5^. The substrate of the hybrid actuator in this work is polymer, which is lighter and less expensive than gold foil. However, the lighter the substrate, the harder to control the geometry of the actuator when immerging it in electrolyte. We still need to optimize the thickness of the polymer layer to get a hybrid cantilevered actuator with tunable geometry and displacement.

## Conclusions

In summary, a novel electrochemical system has been desighed to synthesize nanoporous gold (NPG) and also to drive the NPG based actuator by utilizing a motified rotary TENG driven by human activity. The modified TENG with a cooling system has stable output current and voltage increased by 14% and 20%, respectively, compared to the one without a cooling system. The novel hybrid actuator based on NPG has been driven by the modified TENG with a cooling system. The tip of this cantilever actuator moves as much as 2.2 mm, which is about 10^6^ times larger than that of traditional cantilever using planar surfaces. The energy conversion efficiency during actuation is as high as 56.7% compared to the output of TENG. This light-weight hybrid actuator is promising for applications in Micro-Electro-Mechanical systems such as the driver of rhinoscope, because of its small volume, large displacement, good controllability, low cost and fast response. The hybrid actuator is mainly based on NPG, which is also electrochemically dealloyed by the outputs of TENG with an energy conversion efficiency up to 47%. It is confirmed that more complicated electrochemical activity such as dealloying and actuation can be powered by the modified TENG, which is a stable and sustainable power source.

## Methods

### Fabrication of the Modified TENG

The TENG consists of mainly two parts: a rotator and a stator, both with multilayered structure. The rotator is made up of PMMA substrate and a radially arrayed copper sectors with a central angle of 1° between each sector unit. The stator consists of four components from the top to the bottom: Kapton layer as an electrification material, copper electrode, PMMA substrate and condenser pipe. The circuit printing technology is used for fabrication of the top and bottom grating electrodes, which are composed of two complementary-patterned electrode network. The electrode of stator also formed by a radial array of sectors, with a length about 73 mm, the angle of 1°, and mutually connected at the end. Finally, the diameter of TENG is about 160 mm. A condenser pipe was attached to the bottom of the stator as a cooling system shown in the photograph in Fig. 1(a1).

### Hybrid Actuator Fabrication

A layer of Parylene film with a thickness of 10 μm was deposited on a 10 μm thick Al substrate by thermally activated CVD machine (LH300, La Enterprise) at the pyrolysis temperature of 700 °C. A layer of ca. 120 nm thick gold film was sputtered on the top of Parylene film in Ultra-High Vacuum Magnetron sputtering equipment (Denton Vacuum Discovery 635), then a Au_32_Ag_68_ (at.%) alloy film with the thickness of ca. 350 nm was sputtered on the gold film. Strips with the desired size were cut from the resulting composite foil. Then the Al foil was removed by immersing the strips into 1 M NaOH solution for ca. 1 minute. Thus, the composite materials consisting of polymer, gold film and alloy film was obtained. Then this composite was immersed in 1 M HClO_4_ and connected to TENG circuit for dealloying. The dealloying can be finished in ca. 300 s, after which the hybrid actuator mainly based on NPG was obtained. The NPG microstructure was investigated by the scanning electron microscope (SEM, Hitachi SU8020, Japan). The constituents of the sample before and after dealloying were measured by energy dispersive spectrometer (EDS, IXRF, USA).

### Electrochemical Experiment

All of the TENG’s output electricity was initially transformed and rectified before connecting to the electrochemical circuit. And all of electrochemical procedures in this paper were with two electrodes systems, i.e. reference and counter electrodes were Pt sheet, and working electrode was our sample. The electrolyte used in this work was 1 M HClO_4_ and the ultrapure water (18.2 MΩ, Milli-Q Direct-16, France) was used to prepare the solution and to clean the sample.

## Additional Information

**How to cite this article**: Li, X. *et al.* Nanoporous-Gold-Based Hybrid Cantilevered Actuator Dealloyed and Driven by A Modified Rotary Triboelectric Nanogenerator. *Sci. Rep.* **6**, 24092; doi: 10.1038/srep24092 (2016).

## Supplementary Material

Supplementary Information

Supplementary Video
