# Supplementary material for: Nanoporous-Gold-Based Hybrid Cantilevered Actuator Dealloyed and Driven by A Modified Rotary Triboelectric Nanogenerator
Source: Sci Rep. 2016 Apr 11;6:24092. doi: 10.1038/srep24092 (PMC4827063; doi:10.1038/srep24092)
Supplement: Supplementary Information [file srep24092-s1.doc]

Supporting Information

**Nanoporous-Gold-Based Hybrid Cantilevered Actuator Dealloyed and Driven by A Modified Rotary Triboelectric Nanogenerator**

Xuequan Li^1^, Mengmeng Liu^1^, Baisheng Huang^1^, Hong Liu^1^, Weiguo Hu^1^, Li-Hua Shao^*,1^ & Zhong Lin Wang^* 1,2^

^1^Beijing Institute of Nanoenergy and Nanosystems, Chinese Academy of Sciences, Beijing 100083, China. ^2^School of Material Science and Engineering, Georgia Institute of Technology Atlanta, Georgia 30332–0245, USA. Correspondence and requests for materials should be addressed to L.-H.S. (email: [shaolihua@buaa.edu.cn](mailto:shaolihua@buaa.edu.cn)) or Z.L.W. (email: [zlwang@gatech.edu](mailto:zlwang@gatech.edu)).

The equivalent circuit of a TENG is analogous to a combination of an ideal voltage source Voc with an inner capacitor C_TENG_ as shown in Fig. S1. Then the TENG was linked to a transformer whose ratio of number of turns is 20:1. The AC voltage was rectified by a rectifier after transformation subsequently.

***
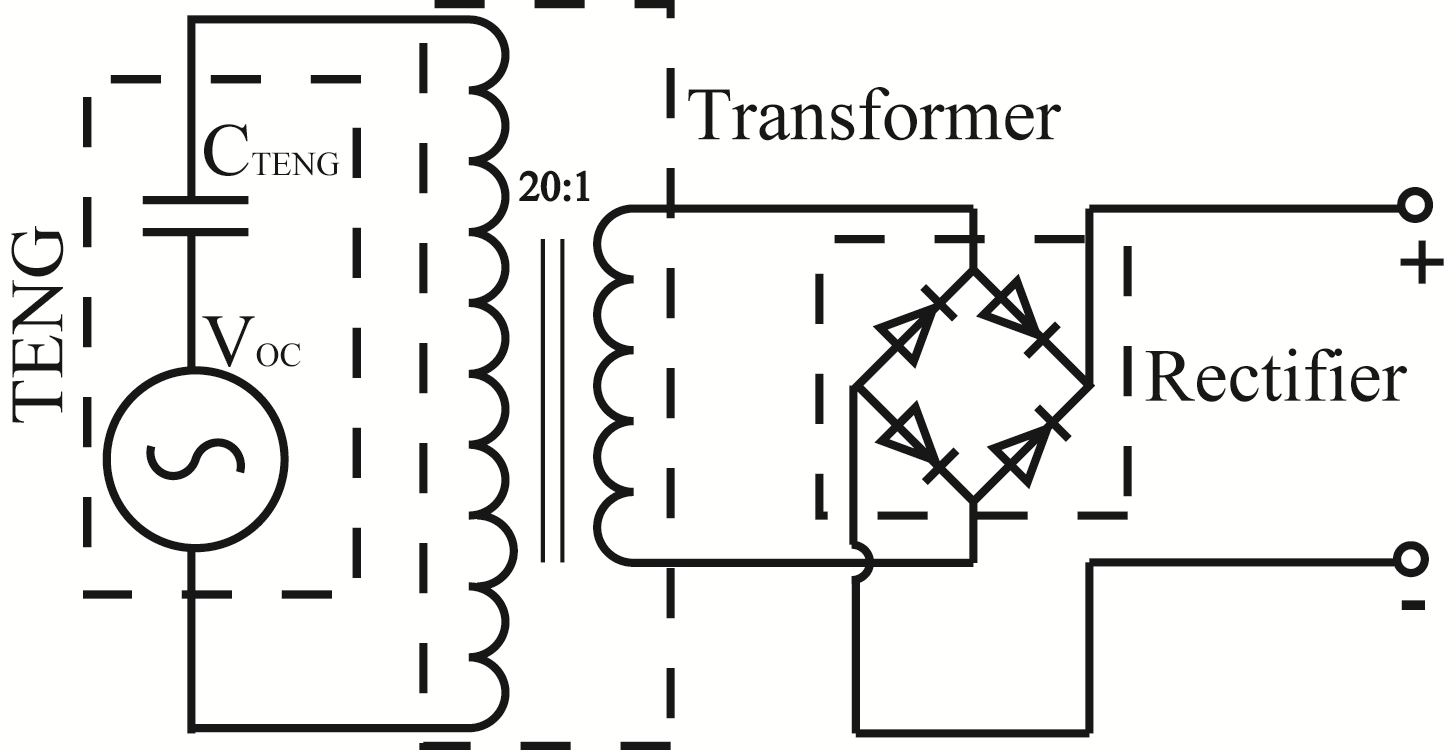
***

**Fig. S1** The equivalent circuit of TENG equipped with a transformer and a rectifier.

**Calculation of the transfered charge and the energy transfer efficiency during dealloying:**

The mean molar mass $\bar{M}$of the Ag_68_Au_32_ alloy:

$\bar{M}=\frac{17}{25}M_{\text{Ag}}+\frac{8}{25}M_{\text{Au}}$=136.38 g/mol

where the molar mass of Ag and Au are $M_{\text{Ag}}$=107.87 g/mol and $M_{\text{Au}}$=196.97 g/mol, respectively.

The electric charge is given by

$Q=F\sum z_{i}n_{i}$, $Q=Fz_{\text{Ag}}n_{Ag}$

where z is the number of electrons tranferred during the reaction and *F* the Faraday constant
*F* = 96485.34 C/mol (coulomb/mole, value from <http://physics.nist.gov/cgi-bin/cuu/Value?f|search_for=faraday> ). Let us assume that Ag dissolves as Ag^+^, *z*_Ag_ = 1.

For Ag_68_Au_32_：

$$Q=Fz_{\text{Ag}}n_{\text{Ag}}=Fn_{\text{t}}x_{\text{Ag}}=F\frac{m_{t}x_{\text{Ag}}}{\bar{M}}=\frac{68}{100}F\frac{m_{t}}{\bar{M}}=\frac{17}{25}96485.34\frac{\text{C}}{\text{mol}}\frac{m_{t}\text{ mol}}{\text{130.14 g}}$$

$Q=\text{504.14 }m_{t}\frac{\text{ C}}{\text{g}}$.

where the total mass of the alloy Ag_68_Au_32_ is *m*_t_ = 0.546mg, thus, Q = 282.32 mC

*W*_work_=*V*_avg_*Q*

*W*_output_ =*V*_avg_*I*_avg_*t*

ζ=*W*_work_/*W*_output_=282 mC/(2 mA*300 s)= 47%

**Calculation of the work done of actuator movement and the energy transfer efficiency:**

$W_{Act}=\frac{F\Delta}{2}$ ; $F=\frac{8EI\Delta}{l^{3}}$; $I=\frac{bh^{3}}{12}$

$$W_{Act}=\frac{\Delta}{2}\frac{8EI\Delta}{l^{3}}=\frac{4E\Delta^{2}}{l^{3}}\frac{bh^{3}}{12}=\frac{Eb\Delta^{2}h^{3}}{3l^{3}}=\frac{3.2\cdot10^{9}\cdot10\cdot10^{-6}\cdot(2.2\cdot10^{-3})^{2}\cdot(2\cdot10^{-3})^{3}}{3\cdot(1.2\cdot10^{-2})^{3}}=239.01\cdot10^{-6}$$

$$W_{Act}\approx0.24\text{mJ}$$

$$W_{small}=\frac{\delta}{2}\frac{8EI\delta}{l^{3}}=\frac{E\delta^{2}}{l^{3}}\frac{bh^{3}}{3}=0.20\text{mJ}$$

*W*_t_ = 2*W*_Act_+ *W*_small_=0.24 mJ*2+0.20 mJ=0.68 mJ

*W*_outputAct_ =*V*_avgAct_*I*_avgAct_*t=*1.2 V﹡0.5 mA﹡2 s=1.2 mJ

ζ=*W*_t_/*W*_outputAct_ = 56.7%

**The program of the single chip to control the output circuit on/off.**

#include <reg52.h>

sbit JD=P3^7;

void main()

{

tt=0;

TMOD=0x01;

TH0=(65536-50000)/256;

TL0=(65536-50000)%256;

EA=1;

ET0=1;

TRO=1;

while(1)

{

if(tt==40)

JD=1; //off

if(tt==80)

{

tt=0;

JD=0; //on

}

}

}

void exter0 interrupt 1

{

tt++;

TH0=(65536-50000)/256;

TL0=(65536-50000)%256;

}
